# Ethnic and Racial Disparities in Clinical Manifestations of Atopic Dermatitis

**DOI:** 10.26502/aimr.0170

**Published:** 2024-06-04

**Authors:** Fihr Chaudhary, Devendra K. Agrawal

**Affiliations:** 1 Department of Translational Research, College of Osteopathic Medicine of the Pacific, Western University of Health Sciences, Pomona CA 91766

**Keywords:** Allergies, Atopic dermatitis, Atopic eczema, Atopy, Erythema, Ethnicity, Filaggrin, Lichenification

## Abstract

Atopic dermatitis is a heterogenous inflammatory skin illness that may last for long time and affect people of different racial and ethnic backgrounds. The condition primarily appears in infants and young children. There are people living with atopic dermatitis in every country and every ethnic group, although the frequency of the disease varies greatly. Due to the varied clinical presentations that atopic dermatitis can have, it can be challenging to characterize and diagnose the disease, particularly in adults. Nevertheless, there exists a dearth of information pertaining to the various presentations of atopic dermatitis among individuals from diverse racial and cultural groups. This critical review article offers a succinct and comprehensive overview of the current findings on the epidemiology of atopic dermatitis with regards to ethnic and racial disparities. The findings hold potential significance in advancing the development of targeted treatments for personalized medicine approaches and enhancing the quality of life for patients with atopy.

## Introduction:

Atopic dermatitis (AD) exhibits erythema accompanied by oedema, vesicles, and oozing lesions during its acute phase, whereas its chronic phase is characterized by the development of skin thickness, known as lichenification [1]. Atopic dermatitis is an often encountered, persistent, and advancing dermatological condition. The prevalence of the condition in affluent nations is estimated to be 15-20% among children and 1-3% among adults [2]. Atopic dermatitis, a skin condition, is influenced by various factors including genetic variables, such as loss-of-function mutations in the filaggrin gene (FLG), as well as environmental factors like climate, nutrition, and lactation [2].

Atopic dermatitis, sometimes referred to as atopic eczema, is a chronic inflammatory skin disorder characterized by recurring symptoms. It exhibits associations with further atopic manifestations, including allergic rhinitis, allergic conjunctivitis, and asthma. AD is the earliest of the atopic symptoms that presents clinical signs and typically begins before the age of 2 years. Patients who are diagnosed with AD have a higher chance of getting additional atopic symptoms later in life. There is a collaborative effort between endogenous and external elements in the manifestation of clinical symptoms of the disease. The presence of exogenous reasons, such as a chilly environment, stress, or pollen, is typically required to develop clinical symptoms, notwithstanding the significance of hereditary factors. Atopic dermatitis is also known as atopic eczema. The word “atopy” originates from the Greek language and means “in the wrong place.” Patients with this illness had a tendency that was distinct from that of normal people; specifically, they tended to become sensitized to environmental influences [3]. Atopic individuals have elevated levels of IgE antibodies in their blood [3].

The diagnosis of AD is primarily based on clinical evaluation, as there are no specific clinical symptoms or laboratory tests that can definitively confirm the presence of the disease. Pruritus represents the primary clinical manifestation of this medical condition, and the distribution of the dermatitis frequently exhibits characteristic patterns, commonly affecting areas such as the face, neck, antecubital fossa, and popliteal fossa. However, the phenomenon of localization exhibits variation across different age groups.

While the clinical diagnosis of AD is the primary method used, accurately defining the condition can be challenging. Consequently, numerous writers have proposed guidelines to assist in the diagnostic process. The diagnostic process relies on an individual’s medical history, encompassing both personal and familial aspects, as well as the identification of characteristic signs and symptoms. Nevertheless, it is important to note that none of these clinical symptoms can be considered diagnostic for AD. There is a lack of precise criteria exclusive to individuals with atopic conditions. Nevertheless, they can contribute to the diagnosis of AD. The age of individuals with atopic conditions appears to have a significant role in the manifestation of several minor criteria, and there are observable ethnic disparities as well. Pruritus is a symptom that exhibits notable resistance to therapy and can be triggered by comorbidities in individuals with atopic dermatitis [3].

During early childhood, it is possible for AD to coexist with seborrheic dermatitis, a condition that can manifest on the face and scalp [3]. Infants frequently experience diaper dermatitis, a condition characterized by skin inflammation in the diaper area. Notably, these infants do not exhibit a familial predisposition to atopic illnesses. Seborrheic dermatitis typically resolves within a few weeks following the cessation of maternal hormone stimulation. Ichthyosis vulgaris may be considered as a potential diagnosis in pediatric patients presenting with excessively dry skin, particularly when there is a positive familial history of ichthyosis. The characteristic occurrence of scaling is particularly observed on the extensor surfaces of the limbs following the attainment of two months of age. The presence of scabies is typically accompanied by intense pruritus and dermatitis. In most cases, additional family members typically exhibit symptoms, and the identification of burrows, commonly seen on the hands, along with the detection of mites, serves as the basis for diagnosis [3]. Psoriasis can occasionally manifest in infancy, albeit infrequently. Subsequently, the lesions exhibit enhanced demarcation and may manifest alongside the characteristic white scale. from childhood and adolescence, AD typically manifests on flexural areas, and it commonly emerges from infancy. Nevertheless, the condition known as atopic winter foot may occasionally be erroneously identified as tinea pedis. There are instances where impetigo affecting the face may be erroneously identified as AD. Psoriasis may also manifest throughout childhood, typically following streptococcal infections in the form of guttate psoriasis. Pityriasis rosea typically initiates with an initial bigger primary lesion, afterwards accompanied by smaller scaly lesions that appear on the trunk. The lesions resolve within approximately one week. Hand eczema in adults may serve as an indicator of AD; nonetheless, it is imperative to rule out potential contact allergens if the eczema persists for an extended period while undergoing therapy. Additionally, there exist infrequent instances in which AD has been documented as a characteristic condition. Nevertheless, the dermatitis observed in these individuals seldom meets the criteria for AD. AD is a prevalent condition that may coexist with other disorders, suggesting the potential presence of comorbidity among affected patients [3].

Atopic dermatitis, a condition primarily impacting the skin, has garnered growing recognition as a systemic ailment due to the accumulation of extensive scientific evidence in recent decades. The pathophysiological state that impacts multiple organs and systems is mostly attributed to the deterioration of the epidermal barrier, chronic inflammation, and IgE-mediated hypersensitivity [4].

## Clinical Cutaneous Manifestations among Different Races

**Figure 1:**
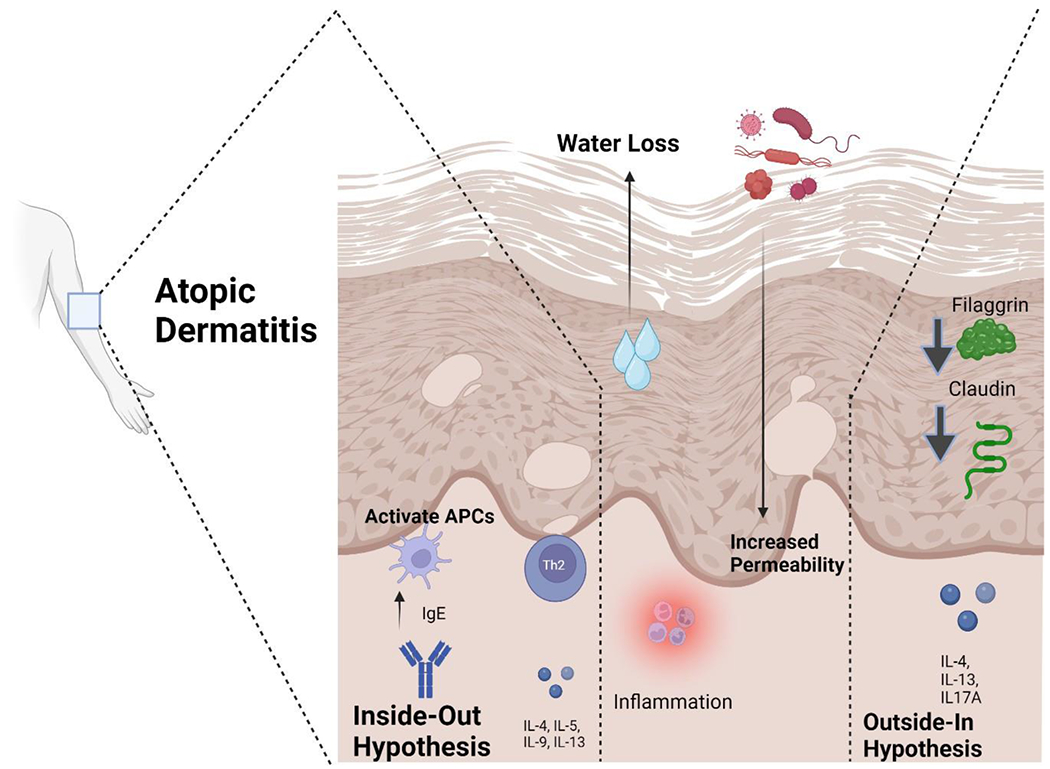
Inside-out and outside-in hypotheses of atopic dermatitis. Several theories have been put out to explain the origins of atopic dermatitis, including as changes to the microbiota, immune system dysregulation, and epidermal barrier malfunction. As a result of these pathophysiological pathways, two main subtypes of AD, known as extrinsic and intrinsic, have been described. The “outside-in hypothesis” has long defined the disease’s extrinsic manifestations, which are caused by a breakdown in the skin’s protective barrier. The dysregulated activity of specific types of immune cells is the driving force behind the intrinsic form, which is also called the “inside-out hypothesis.” APCs - antigen-presenting cells, Th2 - T helper 2 cells, IL- interleukin.

At the epidermal level, the pathogenesis of atopic dermatitis involves the presence of erythema with poorly defined borders, accompanied by pruritus. In the acute stages, vesicles form and then progress to chronicity and liquefaction. Xerotic skin is a recurring condition that frequently affects extensive regions (Figure 1). The correlation between xerosis and pruritus gives rise to the manifestation of post-scratch abrasions, hence augmenting vulnerability to infection. Atopic dermatitis has the potential to impact many sections of the skin, with lesions exhibiting a symmetrical distribution, particularly in areas of the body that are flexible. The shape and distribution of the condition are mostly influenced by the age at which it first appears. In the case of infants, it is predominantly observed on the facial region, neck, scalp, elbows, or knees [5].

## Extra-Cutaneous Clinical Manifestations among Different Races

### Allergic Rhinitis

**Figure 2:**
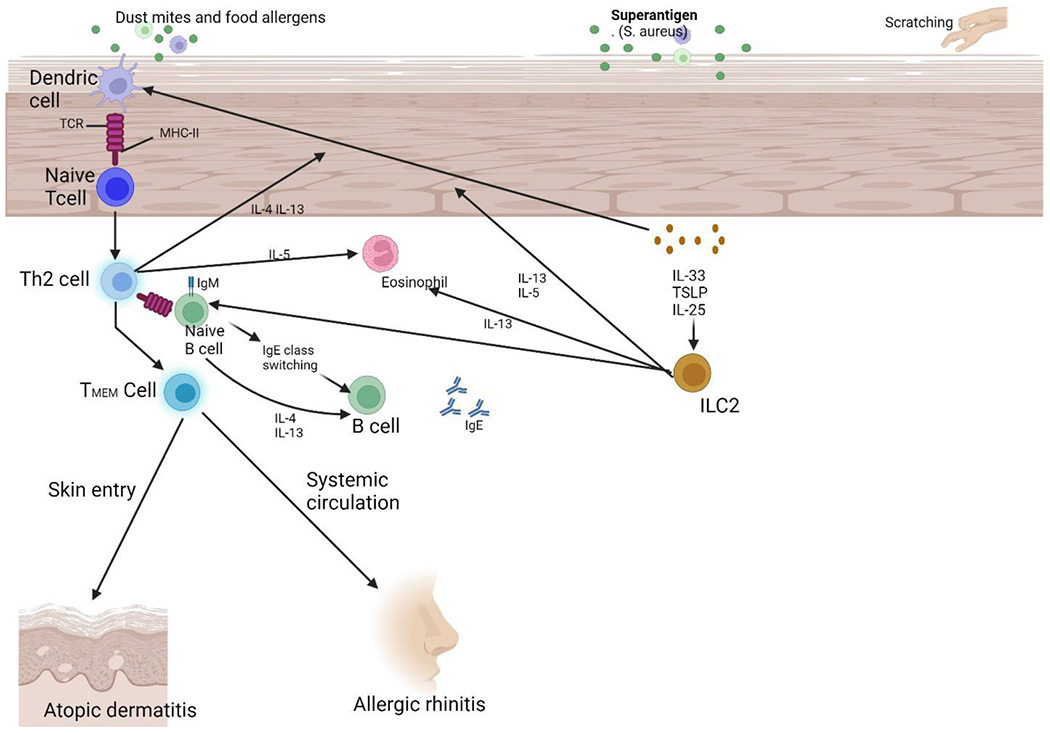
The immunological mechanisms responsible for atopic dermatitis and the development of allergic rhinitis. When skin with a weakened protective layer encounters food allergens, dust mites, or experiences physical damage, it triggers the release of thymic stromal lymphopoietin (TSLP), interleukin (IL)-25, and IL-33 from epithelial cells. These substances then stimulate the activation of immature dendritic cells (DCs) and group 2 innate lymphoid cells (ILC2). Dendritic cells (DCs) that collect allergens travel to lymph nodes where they process the allergens and offer them to naive T cells. This process leads to the production of Th2 cells that are specific to the allergen. Th2 cells exhibit elevated secretion of IL-4 and IL-13 during clonal development and proliferation. They stimulate B cell isotype switching to generate specific IgE cells, hence promoting the synthesis of allergen specific IgE and IgE memory B cells. Allergen-specific IgE attaches to the outer layer of effector cells, such as mast cells and basophils, through the high-affinity IgE receptor (FcεRI). Activation of ILC2 cells intensifies the bias towards Th2 immune response, regardless of the specific antigen. Additionally, this process leads to the formation of memory pools containing allergen specific Th2 and B cells. Allergen-specific Th2 cells with memory circulate and infiltrate the skin, causing a worsening of AD. These cells then enter the systemic circulation and travel to distant organs. Upon further exposure to allergens in individuals who were previously exposed to those same allergens, a variety of atopic illnesses are triggered, leading to the development of atopic march. Abbreviation: TMEM, memory allergen-specific T helper 2 cells.

The phenomenon known as the “atopic march,” which denotes the sequential development of several allergic illnesses, serves as the most definitive evidence supporting the systemic nature of atopic dermatitis [6]. Atopic dermatitis and food allergies often manifest as the primary manifestations of atopy during early childhood. A burgeoning body of data suggests a positive correlation between dermatitis in children and an increased susceptibility to the development of allergic rhinitis and allergic asthma. There is evidence to suggest that dermatitis could potentially serve as an early indicator for the subsequent development of sensitivity to aeroallergens [7]. A notable percentage, potentially reaching 50%, of persons who have been diagnosed with atopic dermatitis eventually manifest asthma, which is subsequently associated with allergic rhinitis [8]. Nevertheless, it is crucial to acknowledge that the perceived level of danger can be exaggerated because the bulk of research studies undertaken on this subject have primarily concentrated on individuals with severe expressions of atopy, a condition that often requires hospitalization. The etiology of atopy is rooted in the progression of immunological hypersensitivity, which is distinguished by elevated levels of immunoglobulin E (IgE) in the bloodstream (Figure 2). The increase in IgE levels is significant in the beginning and perpetuation of allergic reactions, particularly those that impact the skin, respiratory system, and gastrointestinal tract [8].

Allergic rhinitis is a medical condition that results from the complex interaction of genetic and environmental factors. The disorder is characterized by the occurrence of acute or chronic inflammation of the nasal mucosa, which is initiated by an exaggerated immune response to aeroallergens, involving the specific antibodies of the IgE class. Histamine and cysteine leukotrienes are chemical mediators that are generally acknowledged for their participation in the inflammatory response. About 40.5% of persons diagnosed with atopic dermatitis also exhibit symptoms of rhinitis [9]. Patients with early-onset atopic dermatitis exhibited a greater prevalence of rhinitis in contrast to persons who got the illness throughout adolescence or adulthood. Individuals who exhibit atopic symptoms throughout later stages of childhood tend to have milder symptoms because of decreased IgE levels.

The significance of genetic factors in the association between allergic rhinitis and atopic dermatitis was emphasized in a study conducted in Germany. The patients in all cases exhibited a mutation in the filaggrin gene, which was found to increase the vulnerability to rhinitis, regardless of the severity of dermatitis or the age of onset [10]. The clinical presentation of allergic rhinitis includes symptoms such as sneezing, nasal irritation, watery nasal discharge, nasal congestion, diminished olfactory perception, and conjunctivitis, characterized by tears, itching, redness of the conjunctiva, and eyelid swelling. The symptoms are notably widespread among those who exhibit sensitivity to pollen [11]. The presentation of these symptoms in a patient who has received a diagnosis of atopic dermatitis warrants further investigation into probable allergens.

### Allergic Bronchial Asthma

Based on statistical data, a significant percentage of children, ranging from 29% to 35%, who suffer from atopic dermatitis exhibit a susceptibility to developing allergic bronchial asthma [12]. Moreover, previous studies have demonstrated a significant correlation between atopic dermatitis and rhinitis in children, which substantially increases their susceptibility (about 99.3%) to the development of allergic bronchial asthma. Bronchial hyperreactivity plays a crucial role in the development of asthma. It is noteworthy that this condition is also noticed in persons diagnosed with atopic dermatitis. However, it has lately been incorporated into the diagnostic criteria for asthma [12] The experiment indicated above, and its corresponding findings suggest a notable similarity in the pathophysiology of allergic bronchial asthma and atopic dermatitis. Both disorders are predominantly attributed to aeroallergens, and the symptoms of atopic dermatitis can be intensified by either exposure to or direct contact with these allergens. The immunological evidence that substantiates the adverse consequences of respiratory allergen exposure is distinguished by heightened concentrations of antigen specific IgE in the circulatory system. The IgE molecule exhibits binding affinity towards both antigen-presenting cells and allergen-specific T lymphocytes that have undergone activation. A considerable percentage of persons who have been diagnosed with bronchial asthma and atopic dermatitis demonstrated a sensitivity test response rate of 70% when specifically targeting house dust. The clinical manifestation of bronchial asthma comprises a spectrum of variable symptoms, including respiratory limitations, thoracic discomfort, non-productive cough, loud wheezing, and disturbances in sleep patterns [13].

### Food Allergies

**Figure 3:**
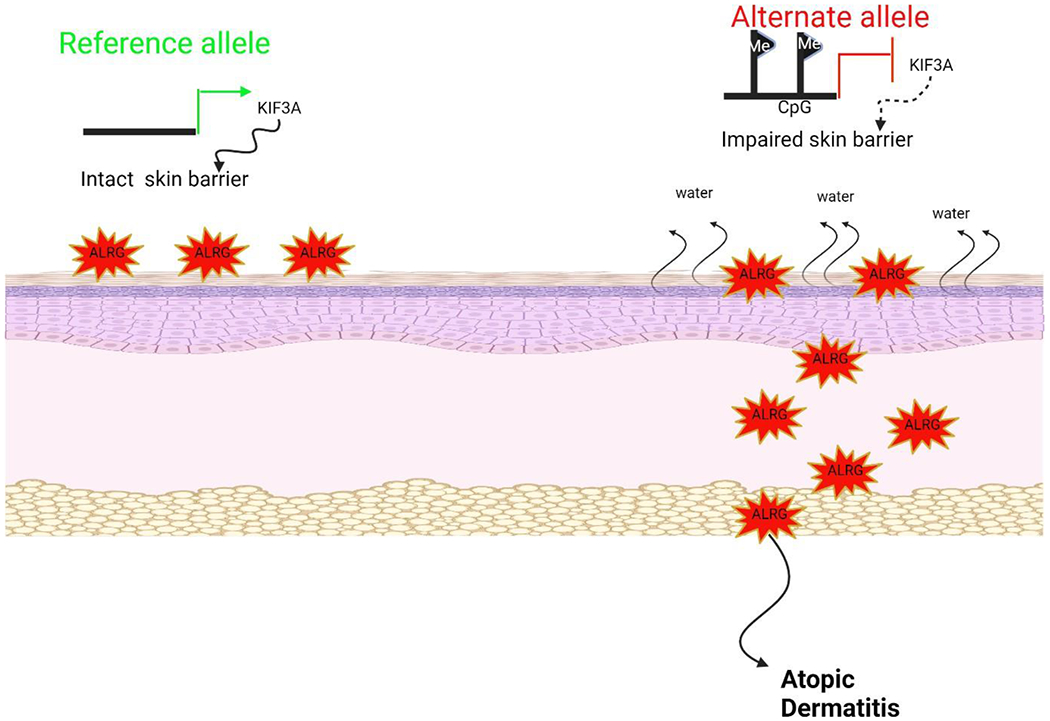
Gene variants elucidate the correlation between a skin disorder and the risk of developing a food allergy. The presence of two prevalent variations in the KIF3A gene heightens the likelihood of early children experiencing a compromised skin barrier and subsequently developing atopic dermatitis. Consequently, this can facilitate the penetration of environmental substances through the skin barrier, thereby contributing to the emergence of food allergies and asthma as individuals mature. This provides further substantiation to an emerging hypothesis that there is a strong correlation between skin health and the well-being of the lungs and gastrointestinal system, a connection that has been underestimated by many. The presence of specific genetic variations in the KIF3A gene is linked to allergic diseases. These variations result in the creation of additional CpG sites, which undergo a high level of methylation in individuals who possess the alternative form of the gene. Consequently, this methylation process leads to a reduction in the production of the KIF3A gene. Reduced levels of KIF3A lead to higher trans epidermal water loss (TEWL) because of impaired cell-cell adhesion, and more vulnerability to the onset of AD. The KIF3A gene plays a crucial role in facilitating the production of primary cilia, which are cellular structures that serve as antennas on the surface of cells. These antennas are responsible for receiving vital signal information from neighboring cells. Similarly, dysfunctions of the identical gene in gastrointestinal tissues can elevate the likelihood of developing food allergies. These allergy risks are linked to a compromised skin barrier, which enables the entry of more allergenic chemicals into our bodies, leading to immune system overreactions. ALRG = allergen.

In recent decades, there have been notable improvements in comprehending the etiology, diagnostic parameters, and therapeutic strategies pertaining to individuals afflicted with atopic dermatitis. A notable proportion, ranging from 20% to 40% of these patients display food allergies [14]. The clinical presentation of these symptoms may exhibit variability depending on factors such as the individual’s age, the extent of coexisting atopic comorbidities, and the responsiveness to therapeutic interventions. The correlation between atopic dermatitis and food allergy is based on various pathological pathways, typically involving processes mediated by IgE or anomalies in type 2 inflammatory immunology (Figure 3). Within the extant corpus of scholarly literature, two discrete concepts have been posited to explicate the correlation between food allergy and atopic dermatitis. One idea suggests that food allergies may play a role in causing or worsening atopic dermatitis in persons who have a preexisting propensity to atopic conditions. On the other hand, the second idea posits that atopic dermatitis may potentially serve as a catalyst for the development of food allergies. The prevailing body of evidence suggests that dietary allergens have a substantial impact on the onset of atopic dermatitis or the worsening of existing symptoms, hence contributing to the development of a more severe presentation of the disorder [15].

A neonate displaying severe atopic dermatitis demonstrates the manifestation of diverse food sensitivities, which can be discerned through the detection of elevated levels of specific IgE antibodies in the bloodstream or by the affirmation of positive outcomes from cutaneous scratch tests (Figure 3). The main dietary allergens commonly linked to atopic dermatitis in babies encompass eggs, dairy products, and nuts. The ingestion of eggs, and to a lesser extent, dairy products, has been associated with the aggravation of symptoms related to eczema [16]. Moreover, nuts have been directly associated with the occurrence of acute allergic reactions, and in more severe instances, anaphylactic reactions.

### Ophthalmic Pathology

While there is a scarcity of research on the correlation between atopic dermatitis and ophthalmic disorders, existing scientific evidence supports the presence of alterations in the ocular surface among individuals with atopic conditions. Among these conditions, the most frequently encountered ones include blepharitis, conjunctivitis, uveitis, keratoconus, and, on rare occasions, detached retinas. A significant proportion, ranging from 25% to 50% of individuals diagnosed with atopic dermatitis experience the development of ocular problems as the condition progresses [1]. Ocular pruritus, which is commonly observed in individuals with atopic conditions, arises from the colonization of germs due to local damage and a decrease in the protective properties of antimicrobial agents on the skin. The prevalence of bacterial colonization in the conjunctival sac and eyelid margins is much higher in individuals with atopy compared to those without this condition, with rates of 86% and 25% respectively. Staphylococcus aureus is identified as the primary causative pathogen responsible for ocular infections in individuals with atopic dermatitis, accounting for around 67% of reported cases [17].

### Digestive Disorders

One of the extra-cutaneous pathologic symptoms of atopic dermatitis is gastrointestinal dysfunction. Serum IgE levels are typically increased, and it is most frequent in children. In addition, samples of duodenal juice from patients with atopy showed elevated levels of reactive immunoglobulins. Increased antigen transport to the impaired intestinal mucosa boosted specific IgE stimulation. This method showed that there may be a connection between gastrointestinal problems and atopy, and that gastrointestinal tract abnormalities may play a role in the etiopathogenesis of atopic dermatitis. Atopy and gastrointestinal morpho-functional problems have been linked before [18]. Eosinophilic gastroenteritis is a condition that primarily affects atopic patients and is defined by the presence of eosinophils in the digestive tract [19]. Common pathogenic factors, such as immunological failure that causes a chronic pro-inflammatory condition, shared genetic abnormalities, and a microbiota imbalance, explain the link between inflammatory bowel illnesses and atopic dermatitis. The cutaneous signs of atopic dermatitis have been linked to alterations in the intestinal microbiome and a lack of diversity in the intestinal microbiota [20].

### Autoimmune Diseases

The association between atopy and autoimmune diseases has been extensively recognized, and recent studies have emphasized the role of atopic dermatitis as a crucial determinant in the onset of autoimmune disorders. There is an increased prevalence of immunological disorders, namely thyroid-related conditions, among pediatric children who have been diagnosed with atopic dermatitis, particularly in cases when lactose intolerance is also present. The existing epidemiological information regarding rheumatoid arthritis, multiple sclerosis, and type I diabetes mellitus suggests that inflammation mediated by Th1 cells may have a protective impact against atopic conditions. Nevertheless, it is crucial to acknowledge that although atopy could mitigate the intensity of autoimmune illnesses, it may not always serve as a preventive measure against their development [21].

Based on the results of meta-analytic investigations, it has been determined that persons who have previously been diagnosed with atopic dermatitis have a notably increased susceptibility to developing alopecia areata, as indicated by a relative risk of 2.5. Moreover, it has been observed that these individuals are also confronted with a significantly higher likelihood of acquiring vitiligo, exhibiting a relative risk of 7.5, in comparison to individuals lacking a past medical record of atopic dermatitis. Therefore, atopic dermatitis has been identified as a potential risk factor for the development of alopecia areata or vitiligo [22].

### Psychological Comorbidities

Atopic dermatitis is characterized as a chronic pathology with periodic outbreaks and remissions and has a detrimental psychological influence on children and adults diagnosed with this condition. Individuals experience the weight of physical affliction, as well as psychological transformations including fluctuations in mood, sleep disturbances, behavioral disorders, attention deficits, anxiety, sadness, and social seclusion, particularly during the stage of adolescence. In addition to the individuals affected by the disease, their families also encounter the economic strain associated with treatment and the challenges of coping with the repercussions of sleep deprivation and the unfavorable social perception of the sickness [7].

Atopic dermatitis is widely acknowledged as a condition that encompasses a psychosomatic element, wherein stress plays a pivotal role in both initiating and perpetuating its flare-ups. The induction of stress has been observed to result in an elevation in the permeability of the epidermal barrier, hence potentially triggering systemic inflammation through the release of proinflammatory neuropeptides. Conversely, the production of neuropeptides in the skin of individuals with atopic dermatitis has been observed to exert an impact on the central nervous system, leading to disturbances in cognitive processes related to recognition and perception, as well as behavioral patterns. The disease’s adverse visual effects serve as a stressor, exerting a significant detrimental psychosocial impact [23].

### Oral Manifestations Associated with Atopic Dermatitis among Different Races

Several cutaneous or systemic illnesses that involve immune processes can present with clinical symptoms in the oral mucosa. Frequently, the initiation of these occurrences takes place within the oral cavity [24]. The relationship between oral lesions and atopy has been previously acknowledged, although the precise mechanisms that contribute to this association remain incompletely understood, and there is a scarcity of research investigating this subject matter. In addition to cutaneous, respiratory, or digestive dysfunction, atopic individuals may develop alterations in the oral mucosa. Individuals with a personal or familial medical history of atopy have been found to exhibit a heightened vulnerability to allergies, elevated levels of serum IgE, and the manifestation of benign migratory glossitis, also known as geographical tongue [25].

An association between geographic tongue and an elevated occurrence of atopy has been shown. However, it is important to note that benign migratory glossitis is not exclusive to atopy and can also manifest in other disorders such as psoriasis, vitamin deficiency, and liver illness [26].

### Epidemiology of Atopic Dermatitis in Diverse Racial and Ethnic Groups

The prevalence of AD demonstrates variability among various nations. There is a notable disparity in the prevalence of eczema between nations in Africa and Oceania, with greater rates observed, and countries in the Indian subcontinent and Northern/Eastern Europe, where the incidence is comparatively lower [27]. The prevalence of eczema in the African population varies from 4.7% and 23.0%. It is worth noting that there is a significant degree of heterogeneity observed within neighboring nations, as well as among cities belonging to the same country. Based on the study, the research findings indicate that the prevalence of AD among children within the age range of 6-7 years in the Asia-Pacific region is estimated to be 17.8% [27]. The prevalence rates exhibit considerable variation among different nations, spanning a range of 4% to 30%.

The fluctuation in prevalence estimates of AD at both the intra- and inter-country levels presents difficulties in ascertaining the influence of ethnicity on the vulnerability to AD. Nevertheless, a study conducted in the United States has revealed a higher prevalence of AD in African American children in comparison to European American children [28]. The National Health and Nutrition Examination Survey conducted during the years 2005-2006 revealed a calculated prevalence rate of 15.6% for AD [29]. The survey findings also indicated that there was a greater prevalence of eczema among African Americans (19.3%) in comparison to European Americans (16.1%) and Hispanics (7.8%). African American (AA) children have a 1.7-fold increased susceptibility to developing AD in comparison to their European American counterparts [28]. The observed connection maintained its statistical significance even when accounting for many confounding variables, including family income, parental education level, geographical location (metropolitan versus rural), and health insurance coverage status. Consequently, children of African descent demonstrate a threefold increased probability of attending a dermatology visit in which AD is diagnosed, although exhibiting significantly lower rates of seeking dermatological treatment. A study conducted in the United Kingdom revealed a significant discrepancy in the occurrence of AD between children of Black Caribbean descent born in London and White children [30]. The results indicated that 16.3% of children of Black Caribbean descent born in London were affected by AD, whereas the prevalence among White children was 8.7% [30], thus revealed that AD is the predominant dermatologic condition diagnosed among children of Black ethnicity.

The disparity in the occurrence of AD between Black and White individuals can potentially be explained by limited availability of healthcare services in Black communities, leading to the presentation of more severe disease stages upon diagnosis [31]. The possible impact of concealing erythema in individuals with darker skin tones on the timely diagnosis or referral to dermatology for severe stages of disease should be considered. Based on a recent longitudinal cohort study conducted over a two-year period, it was revealed that persons belonging to the Black ethnicity demonstrated a decreased probability of disclosing their presence at an office visit for the treatment of eczema. Black patients who sought medical attention exhibited a higher frequency of office visits, greater utilization of prescriptions, and a higher likelihood of consulting a dermatologist for their skin condition compared to individuals of White ethnicity [31].

There is a significant discrepancy in the prevalence of AD diagnoses among Asians and Pacific Islanders in comparison to individuals of White ethnicity [32]. In comparison to those of White ethnicity, Asians and Pacific Islanders exhibit a significantly higher likelihood, almost seven times, of receiving a diagnosis of Atopic Dermatitis during a regular office visit. Nevertheless, the assertion lacks empirical support due to a dearth of extensive population studies. The increased prevalence of AD in Asian populations in comparison to White people can potentially be ascribed, to some extent, to differences in molecular phenotype. The observed alterations encompass a heightened Th17/Th22 polarization and a collective phenotype that displays attributes of both psoriasis and AD [33]. The observation can be made that the incidence of AD seems to be more pronounced in African and Asian nations in comparison to Europe and the United States. In contrast, the occurrence of psoriasis in these geographical areas exhibits a notably diminished prevalence, characterized by a prevalence rate that falls below 0.5%. On the other hand, it has been estimated that the occurrence of psoriasis in the United States and Europe ranges from 2% to 3% [34].

Given the complex characteristics of AD, it appears unlikely that certain gene polymorphisms make a significant impact on the initiation and advancement of AD. Nevertheless, it is widely accepted in the academic community that AD is thought to originate from the collective impact of multiple genes on cellular signaling, immune system activation, and the development of the outermost layer of the skin known as the epidermal membrane [35].

### Overview of Atopic Dermatitis in Different Ethnic Groups

Individuals of Asian descent tend to exhibit lesions that have well-defined borders, which may closely resemble psoriasis plaques [33]. Additionally, these individuals often have greater scaling and lichenification compared to patients with atopic dermatitis who are of Caucasian ethnicity. In contrast, it has been shown that African individuals with AD often exhibit a notable prevalence of extensor involvement [36]. Additionally, these individuals may occasionally display perifollicular accentuation and unique papules on the extensor surfaces and trunk [37]. Moreover, a lichen-planus-like manifestation of AD has been documented specifically in individuals with darker skin types [38].

The presence of loss-of-function mutations (LoF) in the filaggrin structural protein is considered the most significant genetic predisposing factor for the onset of AD. These mutations have been identified in around 50% of European and 27% of Asian individuals diagnosed with AD [39].

It is worth noting that there has been significant research conducted on filaggrin LoF mutations, which have been found in 7-10% of the white European population. However, these mutations are not prevalent in Asian populations, as they exhibit a distinct range of FLG variations [40]. In contrast, previous research has not identified prevalent FLG LoF mutations in individuals of African descent who have AD, despite reports of reduced filaggrin levels in the skin of these patients [41]. It is noteworthy that thus far, no correlation has been established between FLG LoF and AD in African populations, namely those of Ethiopian descent [42, 43]. However, a connection has been reported among African Americans, perhaps attributable to genetic mixing [44]. In general, it has been observed that FLG LoF mutations occur at a significantly lower frequency in individuals of African ancestry compared to those of European and Asian ancestry. This discrepancy implies a relatively smaller role of FLG LoF mutations in the development of AD [45, 46].

The second category of genes associated with vulnerability to AD is connected to the innate and adaptive immune responses, namely the Th2 pathway which plays a crucial role in the development of AD.

Multiple investigations have elucidated a positive association between polymorphisms associated with the Th2 pathway and an elevated susceptibility to developing AD. Genes associated with the immunological response, which are indicative of AD, exhibited certain variations among different ethnic populations, akin to those observed in genes involved in epidermal function. The association between polymorphisms of IL-4 and IL13/IL-13RA1 and a predisposition to AD has been seen in Japanese, Korean, and Chinese populations [47, 48]. Additionally, there is a strong association between AD susceptibility and polymorphisms of IL4RA and STAT6 in Egyptian infants [48]. The IL4Rα gene has a particular polymorphism (Q576R) that has been linked to AD. Notably, this polymorphism has been observed to occur at a higher frequency within the African American community [45]. Additionally, the presence of polymorphisms in the IRF2 gene has been found to be correlated with AD in individuals of European American descent (namely, the rs793814 and rs3756094 variants) as well as in African Americans [49, 50]. Furthermore, a particular variant of the TSLP gene has related to a lower likelihood of persisting AD in both white and African American populations [44].

While the shared characteristics among various ethnic groups outweigh the disparities in all disease aspects, an increasing body of evidence indicates race-specific modifications in the structure of the outermost layer of the skin, as well as variations in the extent of activation associated with distinct immunological pathways. Typically, there may exist notable molecular distinctions in the healthy skin of European, African, and Asian people. The elevated frequency FLG mutations observed in European populations can potentially be explained by evolutionary factors. It is hypothesized that FLG deficiency might have conferred improved immune response against infections, thereby offering protection during historical European pandemics [51]. Additionally, FLG deficiency may have facilitated increased synthesis of vitamin D in the skin, providing an evolutionary advantage in regions with high latitudes [7].

### Presentation of Atopic Dermatitis in Skin of Color

AD symptoms are thought to manifest similarly across cultural groups. The visual appearance of AD is often not thoroughly discussed in the literature, even though the variations in color and distribution of the lesions play a significant role in these subtleties [30].

In AD, pruritic and erythematous plaques with fine overlaying scale commonly manifest on flexor surfaces, particularly the posterior neck, antecubital fossa, and popliteal fossa. Asians had a greater presence of well-defined lesions and a higher incidence of scaling and lichenification in comparison to individuals of White ethnicity [52]. There is a higher prevalence of extensor involvement compared to flexural dermatitis among individuals of African descent [53]. In a study conducted in Nigeria, it was shown that a significant proportion of patients, namely up to 54.1%, exhibited perifollicular accentuation and dispersed discrete papules on their extensors and trunk [52, 54]. Individuals belonging to racial and ethnic minority groups have been observed to display a clinical presentation resembling lichen planus [38].

The incidence of severe AD in Black children is six times greater than that observed in White children [55]. Individuals of African descent are more susceptible to the development of periorbital dark circles, lichenification, and prurigo nodularis compared to individuals of White descent [56]. One possible explanation for this phenomenon is that individuals of African descent who has AD tend to experience a higher incidence of pruritus, leading to increased instances of rubbing and scratching [57].

At the molecular and histological level, there are discernible differences in the manifestation of AD in individuals with ethnic skin, alongside the evident variations in clinical presentation. The observed racial inequalities in epidermal gene expression among individuals with AD may potentially play a role in this phenomenon. The study observed that there was evidence of epidermal hyperplasia, shown by an increase in both epidermal thickness and Ki67 levels, as well as frequent parakeratosis. These findings are typically associated with psoriasis, but are not commonly observed in White patients with AD. The study specifically compared White and Asian AD patients from Korea and Japan [58]. Concomitant with these histological abnormalities, there was a notable increase in Th2 activation, as evidenced by higher gene expressions of markers associated with Th17 and Th22. The association between extrinsic AD, characterized by high IgE levels, and reduced expression of Th17 cells compared to intrinsic AD has been established [58]. Therefore, it is particularly remarkable that out of the 27 individuals of Asian descent included in the study, 26 were found to have extrinsic AD. In general, the cellular and molecular characteristics of Asian AD exhibit a composite pattern that incorporates elements from both European AD and European psoriasis. The therapeutic implications of these findings are noteworthy, since it has been proposed that the targeting of the Th17/IL-23 axis, alongside the Th2 pathway, could potentially yield benefits for patients with Asian atopic dermatitis [58].

### Atopic Dermatitis in Latin America

About 8.5% of the world’s population resides in Latin America (LA), and it is well acknowledged as a region with high rates of social inequality [59]. Disparities in healthcare further complicate the study and treatment of diseases like atopic dermatitis because of other racial/ethnic, geographical, and socioeconomic characteristics present in the area. This section aims to rectify this shortcoming by systematically reviewing studies that examine Atopic Dermatitis in Latin America, including its epidemiology, clinical and laboratory characteristics, ethnic/racial disparities, and treatment interventions.

Results from a study conducted in Bogota, Colombia found that 6.5% of the children evaluated had been diagnosed with AD, and that 42.3% of the children studied had shown dermatological problems [60]. Researchers in Brazil found that 5% of adolescents and 8.2% of school-aged children there suffer from AD on average [61]. A population-based telephone survey was used to determine the prevalence of AD in Brazil [62]. The study determined that the prevalence of AD, when corrected for age, was 2.27 percent [62].

Patients with AD were found to have erythema (54.5%), pruritus (55.1%), and dry skin (48.7%) at their initial assessment [63]. The lesions displayed the classic lichenified or eczematous morphology [64]. Among 1,650 Argentinians with AD, 40% exhibited substantial itching in terms of intensity and frequency [65]. In addition, a whopping 96% said they’d had bleeding or suppuration [65].

A Colombian cross-sectional study found that the condition most affected the flexural regions of the body and frequently coexisted with eyelid dermatitis, hand eczema, and cheilitis [60]. Body surface area (BSA) and the eczema area and severity index (EASI) scores were used to determine the severity of the condition, and most patients were found to have mild to moderate disease [66]. The prevalence of AD in Brazil, Mexico, and Argentina was recently evaluated and the results showed that over half (54.4%) of patients experienced severe pruritus, defined as a Worst Pruritus Numeric Rating Scale (NRS) score of 7 or higher [67]. In addition, nearly half of the 180 people surveyed reported that this severe itching significantly reduced their quality of life [67].

Researchers in Brazil used the Beck Depression Inventory, the Inventory of Stress Symptoms for Adults, and the Dermatology Life Quality Index (DLQI) to assess the mental health of people who had been diagnosed with AD. It was shown that 38.7% of participants showed mild to moderate depression symptoms, while 22.6% showed severe depressive symptoms. In addition, nearly three-quarters (73.3%) showed symptoms of psychological stress, and nearly half (45.2%) reported a significant decline in QoL. Pruritus was found to be a key symptom that contributed to these results [24]. The quality of life for 85.6% of those surveyed in an Argentine study revealed to be negatively affected by AD [65].

Both American and European studies have found that Black children had a higher prevalence of AD. It’s worth noting, too, that there was a large disparity in the overall AD frequency among Los Angeles neighborhoods that were primarily Black. Prevalence rates vary considerably between regions, from 4.4% in Northern Brazil to 10.1% in Cuba [30]. This finding demonstrates that genetic variation does exist amongst African subpopulations. In addition, distinct genes involved in immune regulation and epithelial barrier function contribute to the pathogenesis of AD in certain racial and ethnic groups. Significant contributions to the pathophysiology of AD in European populations have been attributed to loss-of-function mutations in the filaggrin gene [30].

A sizable number of people of European ancestry can be found in the populations of many Latin American countries. Chilean research found that 9.3 percent of their patient group had filaggrin mutations, which are commonly found in European individuals with AD [68]. Filaggrin and claudin-1, a protein involved in tight junctions, were both less abundant in the skin of Brazilian patients. The amounts of these proteins were found to be significantly higher in the conjunctival epithelial cells of people with AD compared to a group of healthy persons, which is interesting. This study indicates that AD-related inflammation may be the cause of the increased protein levels [69]. Environmental elements including temperature, humidity, and sun exposure can all play a part in disease transmission. There is a robust correlation between latitude and AD prevalence in ISAAC Phase 1 [70]. The ISAAC Phase 3 study also found that AD was more common and more severe in facilities that were closer to the Equator [70].

Increased IL-22 expression in AD cutaneous lesions was identified in a Brazilian study [15], which may indicate a switch towards Th22 in AD patients. Higher amounts of IL-17 were found in the serum and skin lesions of people with AD compared to control patients [71]. They also found a reduced CD4 cytokine response after staphylococcal enterotoxin treatment, as well as an increase in IL-22-expressing CD4/CD8 T cells within AD lesions [71].

Emollients washing practices, and avoidance of irritants are generally advised as the first line of treatment in many clinical practice guidelines in Latin America [9]. Primary and secondary therapy options often include topical steroid and calcineurin inhibitor applications [72]. The effectiveness and cost of topical steroids can vary widely, and they have been related to side effects including cutaneous atrophy and adrenal suppression [72]. Using topical corticosteroid inhibitors on particularly sensitive areas including the face and genitalia [72]. However, their high price tag is a major reason why they are rarely used. When topical treatments fail to alleviate severe dermatitis, pharmacological procedures such as phototherapy, oral corticosteroids, and systemic immunosuppressants including methotrexate (MTX) and cyclosporine are used. However, there are unique challenges associated with each of these therapies [72]. Dermatitis patients in Latin America sometimes have trouble accessing phototherapy despite the treatment’s efficacy and safety since they live in rural areas without easy access to phototherapy clinics [73].

## Genetic manifestations of Atopic Dermatitis in Different Racial Groups

### Genetic Differences

AD is commonly acknowledged to exhibit a substantial genetic component, albeit deviating from the conventional features of a monogenetic illness. In contemporary times, genome-wide association studies (GWASs) have broadened their range to include African, Hispanic, and Asian populations, in addition to their earlier emphasis solely on white populations [74]. The study conducted an intensive genome-wide association study (GWAS), which represents the most comprehensive investigation of its kind to date. As a result, the researchers successfully identified 31 risk loci that are significantly related with AD. Variations in genetic markers among several ethnic groups were discovered thus suggesting that the development of AD may be influenced by genes that are related with immunological modulation and epithelial barrier function [74].

The FLG gene has been intensively studied as a prominent genetic risk factor for the onset of AD, with a particular focus on loss-of-function variants. The presence of FLG mutations is often regarded as the most prominent risk factor for AD [75]. The existence of this mutation results in a compromised expression of the filaggrin protein, which is essential for the proper functioning of the skin barrier and the epidermal terminal differentiation process. This encompasses the regulation of pH levels in the skin and the maintenance of sufficient moisture in the outermost layer of the skin [66]. In the study, it was found that there exists a relationship between the presence of FLG loss-of-function mutations and the presentation of more severe and persistent AD, together with an elevated degree of immunological dysregulation [22]. Moreover, people harboring these mutations demonstrate heightened frequencies of cutaneous infections and allergic sensitization. Numerous investigations have consistently documented the existence of loss-of-function mutations in around 50% of European patients and 27% of Asian individuals who have been diagnosed with AD [76].

The occurrence of FLG loss-of-function mutations, such as R501X, 2282del4, S3247X, and R2447X, has been seen to vary between 7% and 10% among individuals of white European descent [77]. Nevertheless, it is important to note that these mutations are generally absent in Asian populations due to the presence of unique FLG null mutations that are exclusive to their ethnic groups. In contrast, another study demonstrated a notable disparity in the prevalence of FLG mutations between African American and European American individuals, with a six-fold variation [78]. Furthermore, a study carried out in Ethiopia failed to reveal any empirical support for a potential association between FLG null mutations or copy number variations and the onset of AD [41, 42]. Generally, it can be observed that FLG mutations are relatively less common in individuals of African descent compared to those of European or Asian descent, despite the higher prevalence of AD in the former population. Therefore, it appears that FLG mutations have a comparatively less deleterious impact on individuals of African descent when compared to those of European or Asian ancestry.

A study that observed changes in polymorphisms of genes associated with innate and adaptive immunity, specifically in the Th2 signaling pathways, among several ethnic groups was conducted [79]. The association between the existence of genetic polymorphisms in the interleukin (IL) 4 receptor a (IL-4Ra) and an elevated susceptibility to both atopy and asthma has been observed [79]. An association between IL-4/IL-4Ra polymorphisms and an elevated susceptibility to AD in the Egyptian population has been shown [47]. A study conducted in 2010 found a correlation between polymorphisms in IL-4 and IL-13/IL13Ra1 and the susceptibility to AD within Korean, Japanese, and Chinese populations [80]. In a mouse model, it has been discovered that a specific polymorphism (Q576R) demonstrates an increased propensity for IL-4Ra-dependent signaling. The observed polymorphism is significantly associated with the development of severe asthma and increased sensitivity to allergens, particularly when mother smoking is present. Moreover, it has been observed that the prevalence of this condition is higher among individuals of African American heritage, with a frequency of 70% in contrast to a mere 20% among individuals of Caucasian ancestry [81].

A specific variant of TSLP is associated with a reduced occurrence of persistent AD in both white and black populations [49]. Furthermore, the research revealed a correlation between polymorphisms in interferon regulatory factor 2 and a heightened susceptibility to AD among the ethnic populations [82]. The high-affinity IgE receptor 1A and Toll-like receptor 2 as indicators expressed on atopic dendritic cells have been characterized, focusing primarily on Japanese people [83]. A positive association between heightened susceptibility to AD and genetic alterations in β-defensin 1, an antimicrobial peptide, across both Mexican and Korean populations has been observed [84]. The researchers reported the identification of single-nucleotide polymorphisms (SNPs) in the Th1 lead cytokine IL-12 and its receptor among persons of Korean heritage who have received a diagnosis of AD [84].

### Differences in the AD Immune Phenotype

**Figure 4:**
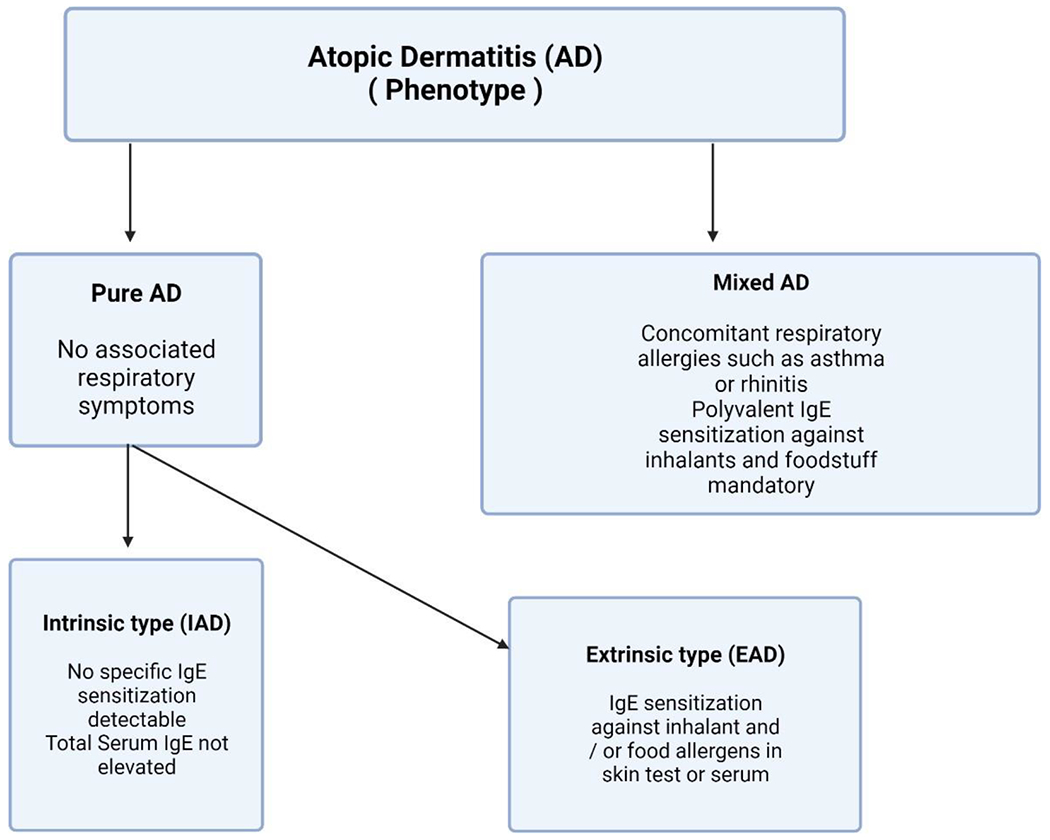
The clinical phenotype of AD can be classified into two primary types: “pure” AD, which does not have any accompanying allergy respiratory illnesses (ARD), and “mixed” type AD, which is characterized by the presence of both AD and ARD. Based on the precise findings of allergologic study using skin tests and specific IgE determinations, “pure” AD can be classified into two subtypes: “extrinsic atopic dermatitis” (EAD) and “intrinsic atopic dermatitis” (IAD).

There are discernible variations in gene expression patterns across individuals with healthy skin from European, African, and Asian populations [85]. These variances potentially have a role in affecting the immunological circuits associated with AD in diverse populations (Figure 4). The primary involvement of T-cells in AD has been initially documented using T-cell targeted treatments, including alefacept, efalizumab, and cyclosporine [86]. The role of the Th2 axis in disease pathogenesis is significant, as evidenced by the clinical effectiveness of the IL-4Ra blocker known as dupilumab. Significantly, the activation of Th2 has been observed in all races and ethnicities that have been studied [87,88].

In addition to the activation of Th2 cells in individuals of European American, African American, Japanese/Korean, and Chinese descent, previous studies have also identified the presence of other Th cell axes in these populations with varying levels of expression [87,88]. Asian patients had a higher degree of Th2 skewing and a lower degree of Th1 skewing compared to European Americans. However, the level of Th2 activation was similar between the two groups. The peripheral blood samples obtained from Asian patients exhibited reduced levels of Th1-related measures (interferon-γ, CCL2, CCL3. CCL4) compared to European Americans. However, both groups demonstrated a shared elevation of Th2 measures (IL-13, CCL13, CCL17, CCL22), with Asian persons showing further increases in CCL26.The number 81 is a positive integer. The observed elevations in Th17 mediators within the skin of individuals of Asian descent diagnosed with AD12 were not observed in the peripheral blood of the same patients. However, levels of IL-22 were found to be higher in serum samples from Asian patients with AD compared to samples from European American patients [89]. This finding aligns with the previously mentioned increases observed in the skin of Asians with AD [88].

### Racial/Ethnic Differences in Incidence and Persistence

In a longitudinal study involving a cohort of 1,437 children, it was discovered that children belonging to non-Hispanic black racial background and other non-Hispanic races exhibited a greater propensity for developing incident AD during the early stages of infancy. On the other hand, there was no significant difference in the likelihood of developing AD between Hispanic children and non-Hispanic white children. Non-Hispanic black children and Hispanic children demonstrated elevated likelihoods of suffering AD in comparison to non-Hispanic white children.

Previous studies have investigated the frequency and intensity of AD during childhood, with a particular emphasis on differences related to race and ethnicity. Numerous research studies have consistently indicated that children from non-white racial backgrounds have a higher prevalence and more severe symptoms of AD in comparison to their white counterparts [90,91].

The findings of a cohort research conducted in the United States indicate that white children had a higher probability of developing persistent AD in comparison to non-white children [92]. Furthermore, a separate study carried out in the United States showed that children of non-white ethnicity had higher probabilities of experiencing persistent AD when compared to their white counterparts [93].

### Association of Filaggrin Loss-of-Function Variants with Race in Children with Atopic Dermatitis

**Figure 5:**
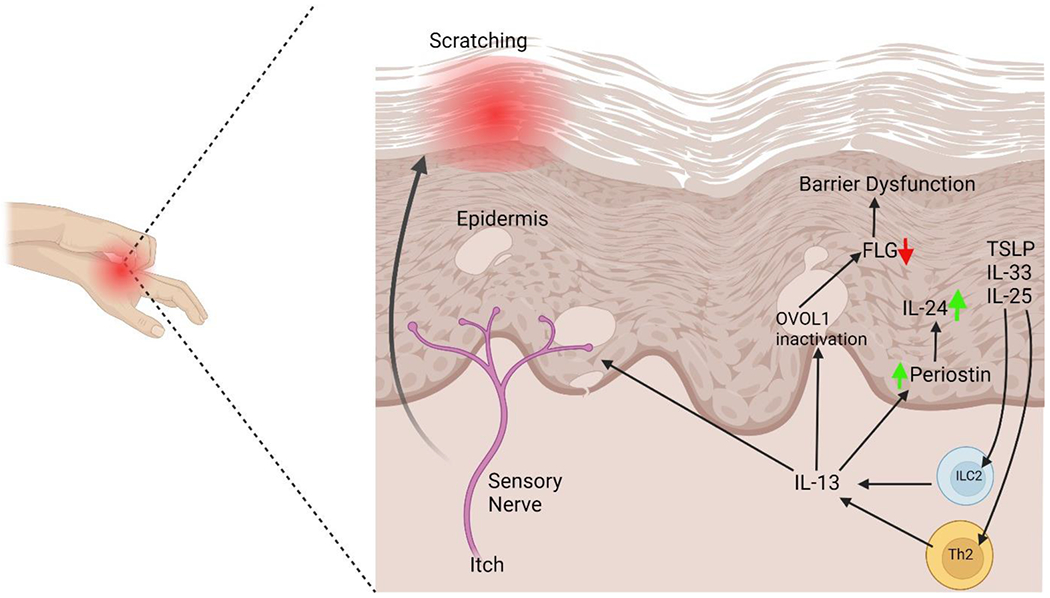
Streamlined mechanism of filaggrin (FLG) mutations and atopic dermatitis development. Th2 cells and ILCs in the affected skin of atopic dermatitis patients generate substantial quantities of interleukin-13 (IL-13). IL-13 causes the inactivation of OVOL1 and increases the activity of the periostin-IL-24 axis, resulting in the downregulation of filaggrin (FLG) and the subsequent impairment of the skin barrier. Barrier failure increases the production of thymic stromal lymphopoietin (TSLP), IL-25, and IL-33 in the epidermis. These cytokines enhance the process of Th2 cell differentiation and the development of group 2 ILCs (ILC2), hence facilitating their production of IL-13. IL-13 additionally activates the sensory nerve and elicits the sensation of itch. Scratching caused by itching exacerbates the impairment of the skin’s protective barrier.

Exon 3 loss-of-function (LoF) variations in the filaggrin gene (FLG [OMIM 135940]) are frequently cited as the genetic variants most frequently linked to skin barrier malfunction (Figure 5). There are thirteen FLG codes that correspond to a protein responsible for maintaining the integrity of the skin barrier. It has been observed that FLG LoF variations are linked to the occurrence and continuity of AD [94, 95]. FLG LoF variants are prevalent in around 25% to 30% of individuals of European and Asian descent who have AD. However, except for two limited case series, individuals of African ancestry with AD rarely demonstrate FLG LoF variations. The presence of 13, 14, and 20-22 FLG LoF variants has been observed in persons who do not have AD. Notably, these variations are prevalent in approximately 8% of the general European population [96]. The gnomAD (genome aggregation database), a globally collaborative collection of exome and genome sequencing data, has revealed around 500 FLG LoF variations. This data is supported by the Broad Institute located in Cambridge, Massachusetts. Multiple investigations have demonstrated variations in the occurrence of FLG LoF mutations among different racial groups [97,98,99,100,101,102].

There is a persistent association between four FLG LoF variants (p.R501*, c.2282del4 [p.S761fs], p.S3247*, and p.R2447*), and Atopic Dermatitis in individuals of European descent [43,44]. These variations have allelic frequencies ranging from 1% to 10%. Consequently, much research largely concentrates on investigating the impact of these specific variants. Nevertheless, a comprehensive examination of persons of East Asian descent afflicted with AD27 revealed that a more extensive range of genetic variations must be analyzed to thoroughly assess the potential correlation between FLG LoF polymorphisms and the prevalence of AD. Previous research employed various genotyping and sequencing methods and relied on an earlier bioinformatics pipeline, however, failed to identify loss-of-function variants in the FLG gene among individuals of African descent with AD at the same frequencies observed in European and Asian populations [96,80,103]. Nevertheless, current research utilizing more advanced analytical methods has revealed that specific FLG LoF variants can be found in individuals of African descent who have AD. However, the prevalence of these variants is below 2%, indicating the necessity for a more extensive evaluation with a bigger sample size and a greater number of variants.

A thorough compilation of loss-of-function (LoF) variations in the FLG gene among a cohort of children in the United States who have mild to moderate AD has been provided [43]. The observed variations exhibit significant racial disparities in their impact on the prevalence of AD. The disparity between races can have a significant impact, as certain genetic variations that are prevalent in one race may be scarce or completely absent in another. Furthermore, the specific positioning of these variations within exon 3 might exhibit substantial variation across different racial groups. In general, there is a higher prevalence of FLG LoF variants among white children with AD compared to African American children, with the former being more than twice as likely to possess such variants. Additionally, African American children tend to exhibit a higher prevalence and persistence of AD.

## Atopic Dermatitis in the Developing Countries of Asia, Africa, Latin America, and the Middle East

The incidence of AD appears to be increasing in developing nations [104], potentially attributable to factors such as urbanization, pollution, adoption of Western foods, and obesity [104]. The International Study of Asthma and Allergies in Childhood (ISAAC) Phase 3 revealed a notable prevalence of AD in adolescents aged 13 to 14 years in Africa and Latin America [105]. On the other hand, it is worth noting that Asian-Pacific countries, the Eastern Mediterranean region, and the Indian subcontinent exhibited comparatively lower prevalence rates of AD, which ranged from 3% to 6% [106]. The prevalence of AD among children aged 6 to 7 years was found to be high in Asian-Pacific countries, Africa, and Latin America, with rates estimated to be about 10% [106]. Nevertheless, it is worth noting that the prevalence rates of AD in the Eastern Mediterranean region and the Indian subcontinent were comparatively lower, ranging from 3% to 5% [105]. Similarly, the occurrence rate during the initial two years of life was found to be noteworthy, ranging from 7% to 27%, in Asian-Pacific countries such as South Korea, China, Singapore, Malaysia, and Taiwan [107].

In general, AD manifests in a comparable manner among individuals of different racial and ethnic backgrounds, characterized by persistent or recurring itchy skin lesions with eczematous qualities. Nonetheless, certain aspects of the condition may exhibit varying degrees of prominence in those with darker skin. Individuals with darker skin may exhibit less pronounced erythema, which can manifest as a violaceous hue, rather than the typical red coloration [108]. Individuals with darker skin may have heightened manifestations of perifollicular accentuation, papulation, scaling, lichenification, and pigmentary alterations [91]. Moreover, it has been observed that Asian populations exhibit a higher degree of polarization towards helper T cell (Th) 17/Th22, in comparison to other groups [88].

The polarization pattern gives rise to a phenotype that encompasses characteristics of both AD and psoriasis [62]. The distribution of lesions may exhibit variation, particularly among patients with darker skin tones, who are more prone to developing lesions on extensor surfaces rather than the conventional pattern of primarily flexural lesions. Variations in the age at which an illness manifests, the severity of the disease, and the genetic predisposition to the disease may be observed among different racial and ethnic populations. The occurrence of Atopic Dermatitis (AD) in adult individuals seems to be more prevalent within some Asian groups, possibly due to elevated rates of disease commencement throughout adulthood. Despite the decreased prevalence of the two most prevalent filaggrin (FLG) mutations associated with AD, individuals of African origin seem to face an increased susceptibility to severe forms of AD. Previous research has documented the occurrence rates of various FLG mutations among individuals of Asian and Middle Eastern descent. According to Hajdarbegovic and Thio (2012), those with darker skin may experience a heightened severity of pruritus [109].

## Age and race do matter!

Even though the cliché “children are not small adults” is frequently used, most of the immunologic research on AD is conducted on adults. This information is unlikely to provide insights into the early events that trigger AD in newborns and young children, who represent the primary age group affected by this condition. A study was conducted to examine the cytokine pathway activation in polarized, activated, circulating cutaneous lymphocyte antigen (CLA)1 skin-homing T cells of children and adults with AD [110]. The study observed a considerable expansion of CLA1 Th2-cell counts in both pediatric and adult individuals with AD, as compared to control participants. However, it was found that children with AD had significantly lower numbers of CLA1IFN-γ1 Th1 cells in comparison to adults with AD [78,89]. In contrast to the adult population, no discernible imbalances were observed in the CLA2 T cell subset among pediatric patients with AD. Similarly, no significant deviations in the frequency of Th22 cells were discovered in this patient group [110]. On the other hand, individuals classified as adults with AD had a higher occurrence of CD4+ and CD8+ T cells that produce IL-22 within the population of T cells that migrate to the skin. The data presented in this study indicates that the activation of Th2 cells that migrate to the skin may be a contributing factor in the development of AD [79,89,110]. In addition to variations associated with age, it is increasingly recognised that there exist racial disparities in the manifestation of phenotypic characteristics connected to Atopic Dermatitis. A study which examines and compares the histologic and genomic characteristics of AD in two distinct populations: European Americans and Eastern Asians (specifically, Japanese and Korean individuals) by obtaining the lesional skin biopsy specimens from both groups of individuals with AD exhibited characteristics such as epidermal hyperplasia, infiltration of T-cells and dendritic cells, and polarization towards Th2 immune response found notable distinctions were identified [78,89].

Asian patients exhibited a histologic appearance that resembled psoriasis, characterized by heightened elongation of epidermal rete ridges and the presence of parakeratosis, which are common features observed in individuals diagnosed with psoriasis. Although both groups exhibited elevated IgE levels, skin biopsy samples from Asian patients with AD demonstrated notably higher expression of Th17- and Th22-related cytokines (specifically, IL-17A, IL-19, and IL-22) as well as IL-17/IL-22 compared to skin biopsy samples from European American patients. The observed results could perhaps explain the heightened occurrence of psoriasiform alterations in Asian individuals with AD, as both IL-19 and IL-22 have been established as inducers of epidermal hyperplasia and parakeratosis. It is noteworthy that the induction of IL-19 can be attributed to both IL-17 and IL-4/IL-13 [94]. The coactivation of Th2/Th17 in Asian patients diagnosed with AD exhibits notable differences compared to European American individuals with AD, as well as dissimilar from the primary activation of Th17 observed in patients with psoriasis. Future clinical trials using pathway-selective antagonists will be necessary to determine the relative pathogenic contributions of the Th2 and Th17 axis in Asian individuals with AD. The immunological activation pathways in individuals of African American descent diagnosed with AD have yet to be elucidated. Individuals with intrinsic AD have higher levels of IL-17 compared to patients with extrinsic AD [111].

## African American ancestry contribution to atopic dermatitis

Despite notable advancements in treatment strategies and an improved understanding of environmental risk factors, there are considerable racial inequalities in the prevalence of AD that cannot be completely ascribed to non-genetic reasons. The presence of inequities is additionally compounded by an unjustifiable absence of participation of minority populations in genetic and pharmacogenetic studies related to asthma and AD. As a result, the comprehension of genetic risk factors for several clinical disorders in communities of African heritage is notably less thorough as compared to people of European descent. The presence of shared genetic foundations of disease among African heritage communities and other population groups is likely attributed to their distinct evolutionary histories and differential amounts of environmental exposure. Nevertheless, it is plausible that unique genetic risk factors play a role in the higher occurrence and intensity of asthma and AD in individuals of African ancestry. This assertion is substantiated by the existence of diverse illness characterization indicators and variances in the efficacy of medicinal therapies employed for various disorders. Previous studies have employed admixture mapping as a means to identify disease-associated genes in African Americans, specifically for conditions like prostate cancer and kidney disease [112]. However, there is a dearth of documented successful admixture mapping studies in the existing literature that specifically investigate the relationship between asthma and AD in African Americans. This implies that the studies in question may have produced insignificant results, either due to publication bias, or that researchers studying asthma and AD have not extensively employed this methodology.

## Temporal and Racial Differences Associated with Atopic Dermatitis Staphylococcus aureus and Encoded Virulence Factors

AD is a dermatological disorder characterized by inflammation of the skin, which has a significant correlation with the presence and proliferation of Staphylococcus aureus, a bacterium known for its colonization and infectious properties. Staphylococcus aureus strains undergo periodic shifts in populations approximately every ten years, which are contingent upon the presence of specific virulence factors. The observed variations in the prevalence and severity of AD among different racial groups could be partially attributed to alterations in the virulence factors of Staphylococcus aureus. A total of 103 S. aureus isolates obtained between 2011 and 2014, together with 100 isolates from 2008, were subjected to analysis to determine the prevalence of genes responsible for encoding superantigens (SAgs).

According to a study conducted in 2008, the prevalence of AD among youngsters exceeded 10% [113]. Additional examination of host race reveals a notable disparity in prevalence rates among African American and European American children, with percentages of 15.9% and 9.7% correspondingly [113]. Several studies have been conducted to investigate the association between the prevalence of AD and variations in disease severity among individuals of different racial backgrounds. These studies have examined differences in the composition of stratum corneum ceramides, transepithelial water loss (TEWL) [114], pH levels [115], mutations in the filaggrin gene [64], and the presence of Staphylococcus aureus in the nasal passages. None of these factors in isolation can be solely attributed to the different presentation of Atopic Dermatitis in African American (AA) versus European American individuals. This implies that there may be additional factors at play that account for the observed disparities. It is noteworthy that a study revealed the presence of racial disparities in immunological activation among individuals diagnosed with Atopic Dermatitis [78]. Staphylococcus aureus has been seen to be present in 40 to 100% of AD lesions, with bacterial counts reaching as high as 107 colony-forming units per square centimeter [116]. The data reported in a parallel study indicates that there are notable distinctions among the S. aureus strains that infect individuals of African American (AA), European American (EA), and Mexican American (MA) descent [117]. Variations in the occurrence and intensity of diseases may arise because of this.

## Conclusions

Atopic dermatitis is a skin disease affecting people of different racial and ethnic backgrounds, characterized by erythema, oedema, vesicles, and seeping lesions in its acute stage and skin thickness in its chronic stage. It affects 15-20% of children and 1-3% of adults in wealthy countries. The disease is primarily based on clinical evaluation, with pruritus being the primary clinical manifestation. The diagnosis relies on an individual’s medical history, personal and familial aspects, and the identification of characteristic signs and symptoms. Atopic dermatitis can coexist with other conditions, such as seborrheic dermatitis, diaper dermatitis, and ichthyosis vulgaris in early childhood. Atopic dermatitis is primarily affecting the skin and has been recognized as a systemic disease. Clinical cutaneous manifestations include erythema with poorly defined borders, pruritus, vesicles, xerotic skin, and a symmetrical distribution of lesions. Extra-cutaneous clinical manifestations include allergic rhinitis, a medical disorder arising from the interplay between hereditary and environmental variables. Allergic rhinitis is distinguished by acute or chronic inflammation of the nasal mucosa, triggered by an excessive immune response to aeroallergens, involving histamine and cysteine leukotrienes. A German study highlighted the importance of hereditary variables in the connection between allergic rhinitis and atopic dermatitis.

Atopic dermatitis is a common condition that can coexist with other disorders, suggesting potential comorbidity among affected patients. Allergic bronchial asthma is a common condition in children, with a significant proportion prone to developing it. Food allergies are associated with atopic dermatitis, with approximately 20-40% of patients exhibiting these symptoms. In infants, severe atopic dermatitis can lead to food sensitivities, with eggs, dairy products, and nuts being primary food allergens. Ophthalmic pathology, such as blepharitis, conjunctivitis, uveitis, keratoconus, and detached retinas, are common among individuals with atopic conditions.

Atopic dermatitis is also linked to autoimmune diseases, with the incidence of thyroid-related disorders being elevated in pediatric patients diagnosed with atopic dermatitis. Atopic dermatitis is a chronic pathology with periodic outbreaks and remissions, causing significant psychological and physical affliction for both individuals and their families. Oral manifestations include cutaneous, respiratory, or digestive dysfunction, alterations in the oral mucosa, heightened vulnerability to allergies, elevated IgE serum levels, and benign migratory glossitis. AD is a common condition with a significant disparity in diagnosis rates between Asians and Pacific Islanders compared to Whites. Asians and Pacific Islanders are seven times more likely to receive a diagnosis of atopic dermatitis during a routine office visit compared to individuals of White ethnicity. This may be due to variations in molecular phenotype, such as a more pronounced Th17/Th22 polarization and an overall phenotype that exhibits characteristics of both psoriasis and atopic dermatitis. Loss-of-function mutations (LoF) in the filaggrin structural protein are considered the most significant genetic predisposing factor for the onset of atopic dermatitis. However, these mutations are not prevalent in Asian populations, as they exhibit a distinct range of FLG variations. The second category of genes associated with vulnerability to atopic dermatitis is connected to the innate and adaptive immune responses, namely the Th2 pathway, which plays a crucial role in the development of atopic dermatitis. Race-specific modifications in the structure of the outermost layer of the skin and variations in the extent of activation associated with distinct immunological pathways may explain the elevated frequency of FLG mutations observed in European populations.

Atopic dermatitis symptoms are thought to manifest similarly across cultural groupings, with differences in pigmentation and distribution of lesions accounting for most of the subtleties in the visual appearance of atopic dermatitis. Black children have a six-fold higher risk of severe AD than White children, possibly due to more pruritus and rubbing and scratching. Latin America, comprising 8.5% of the global population, is characterized by significant social inequality and healthcare disparities. Atopic dermatitis is a significant issue in Latin America, with patients exhibiting flexural lesions in the popliteal and antecubital regions, as well as dispersed on extensor surfaces such as the extremities and trunk. The primary manifestations observed in patients with atopic dermatitis are erythema (54.5%), pruritus (55.1%), and dry skin (48.7%). The presentation of AD symptoms in different cultural groups is complex and multifaceted. Understanding the underlying genetic, environmental, and clinical factors can help develop targeted treatments and interventions to improve the quality of life for individuals with atopic dermatitis. A study in Brazil found that 38.7% of individuals diagnosed with atopic dermatitis exhibited moderate to severe depressive symptoms, while 22.6% had severe depressive symptoms. atopic dermatitis is a genetic disorder with significant genetic differences among different ethnic groups. Genome-wide association studies (GWASs) have expanded their scope to include African, Hispanic, Asian, as well as white populations. Loss-of-function mutations in the filaggrin gene have been identified as significant contributors to the pathogenesis of atopic dermatitis in European populations. T-cell targeted treatments, such as alefacept, efalizumab, and cyclosporine, have been initially documented, but the role of the Th2 axis in disease pathogenesis is significant.

A study involving 1,437 children found that non-Hispanic black and non-Hispanic children had a higher likelihood of experiencing atopic dermatitis during early infancy, while Hispanic children had comparable odds of acquiring atopic dermatitis. Non-white children also had higher probabilities of experiencing chronic attention deficit compared to non-Hispanic white children. Previous research has consistently demonstrated that nonwhite children experience a higher incidence and greater severity of atopic dermatitis compared to their white counterparts. Exon 3 loss-of-function variants in the filaggrin gene (FLG) are frequently linked to skin barrier malfunction, with FLG LoF variants prevalent in around 25% to 30% of individuals of European and Asian descent who have atopic dermatitis. However, African American children have a higher prevalence and persistence of atopic dermatitis compared to white children. Atopic dermatitis is also prevalent in developing countries like Asia, Africa, Latin America, and the Middle East due to factors such as urbanization, pollution, Western diet consumption, and obesity.

Atopic dermatitis manifests similarly among individuals of different racial and ethnic backgrounds, characterized by persistent or recurring itchy skin lesions with eczematous qualities. Asian populations exhibit a higher degree of polarization towards Th17/Th22 cells, giving rise to a phenotype that encompasses characteristics of both atopic dermatitis and psoriasis.

In summary, atopic dermatitis is a complex and diverse skin disease that affects people of different racial and ethnic backgrounds. While the clinical presentation may vary, genetic and environmental factors play crucial roles in the development of this condition. Understanding these differences is essential for developing targeted treatments and interventions that can improve the quality of life for individuals with atopic dermatitis. Additionally, there are notable disparities in healthcare access and outcomes among different racial groups, emphasizing the need for addressing these issues to provide better care for all patients affected by atopic dermatitis.
